# Susceptibility, Immunity, and Persistent Infection Drive Endemic Cycles of Coxiellosis on Dairy Farms

**DOI:** 10.3390/ani14071056

**Published:** 2024-03-29

**Authors:** Jens Böttcher, Michaela Alex, Sven Dänicke, Jörn Gethmann, Katja Mertens-Scholz, Britta Janowetz

**Affiliations:** 1 Bavarian Animal Health Service, Senator-Gerauer-Straße 23, D-85586 Poing, Germany; michaela.alex@tgd-bayern.de (M.A.); britta.janowetz@tgd-bayern.de (B.J.); 2 Institute of Animal Nutrition, Friedrich-Loeffler-Institute, Federal Research Institute for Animal Health, Bundesallee 37, D-38116 Braunschweig, Germany; sven.daenicke@fli.de; 3 Institute of Epidemiology, Friedrich-Loeffler-Institute, Federal Research Institute for Animal Health, Südufer 10, D-17493 Greifswald-Insel Riems, Germany; joern.gethmann@fli.de; 4 Institute for Bacterial Infections and Zoonoses, Friedrich-Loeffler-Institute, Federal Research Institute for Animal Health, Naumburger Straße 96a, D-07743 Jena, Germany; katja.mertens-scholz@fli.de; 5 Institute for Infectious Diseases and Infection Control and Center for Sepsis Care and Control (CSCC), Jena University Hospital, Am Klinikum 1, D-07745 Jena, Germany

**Keywords:** Q fever, *Coxiella burnetii*, cattle, dynamics of infection

## Abstract

**Simple Summary:**

Dairy farms are frequently endemically infected with *Coxiella (C.) burnetii*, the etiological agent of coxiellosis. In human medicine, phase (Ph)-specific tests are used to differentiate serological responses in acute and chronic infections. In our study, we adapted this paradigm to milk testing in dairy cows in order to better understand the dynamics of coxiellosis in dairy farms. Some cows develop a persistent infection, which is characterised by the shedding of *C. burnetii* into milk or by amniotic fluid at calving, and have increased PhI antibody titres (≥100). These cows subsequently serve as a source of infection for susceptible young cows. Recently infected young cows then start to shed *C. burnetii* at calving, thus slowly increasing the infectious environmental pressure, and as *C. burnetii* is primarily shed at calving, it results in an early infection of calves. Calves subsequently develop cellular immunity without detectable antibodies, which is typical for intracellular pathogens. At the time when these animals enter the cow herd two years later, the highest level of herd immunity is reached. At this time, shedding at calving ceases, and new young, susceptible animals enter the herd. The risk of a new infectious cycle now increases with the number of susceptible young cows and their subsequent infection by persistently infected older cows. This dynamic infectious process is mirrored by a wave-like pattern of phase-specific antibody profiles that differ by age groups. Seronegative age groups indicate recent time periods of high-level shedding at calving and are flanked by seropositive age groups, which experienced primary infection in adulthood. Persistently infected cows are generally detected in the older cow group. A serological PhI^−^/PhII^+^ pattern in first lactation indicates an ‘acute’ state of herd infection, while a PhI^+^/PhII^+^ pattern indicates a ‘chronic’ state of herd infection. No detectable antibodies in primiparous cows represents a ‘silent’ state of herd infection if antibodies are detected in older cows; otherwise, the herd is assumed to be free of coxiellosis.

**Abstract:**

*Coxiella (C.) burnetii*, a zoonotic bacterium, is prevalent in dairy farms. Some cows develop a persistent infection and shed *C. burnetii* into milk and occasionally by amniotic fluid at calving. Serological diagnosis of Q fever in humans is performed by phase (Ph)-specific antibody tests; PhII antibodies usually indicate an acute infection, while the development of a chronic infection is characterised by elevated PhI antibody titres. Phase-specific tests have now been established for diagnosis of coxiellosis in cattle. Additionally, an interferon-γ (IFN-γ) recall assay has been implemented to assess cellular immunity to *C. burnetii* in cattle. Milk samples from all lactating cows (n = 2718) of 49 Bavarian dairy farms were collected through a convenience sample and analysed for phase-specific antibodies. Antibody profiles were evaluated by age. Based on the seropositivity of first-lactation cows, three distinct herd profiles were observed: an ‘acute’ state of herd infection was characterised by a PhI^−^/PhII^+^ pattern. The detection of PhI antibodies (PhI^+^/PhII^+^) characterised the ‘chronic’ state, and seronegative results defined the ‘silent’ state of herd infection. If antibodies had not been detected in multiparous cows, the herd was considered as probably free of coxiellosis. The analysed cattle herds were noted to have an ‘acute’ (n = 12, 24.5%), ‘chronic’ (n = 18, 36.8%), or ‘silent’ state of herd infection (n = 16, 32.6%). Only three farms (6.1%) were classified as ‘free’ of *C. burnetii*. The detection of these herd states over a time period of 4 years in one farm indicated that the described states occur in a cyclical manner. Frequently, a wave-like profile was seen, i.e., a circumscribed seronegative age group was flanked by seropositive age groups. In seronegative animals, IFN-γ reactivity was demonstrated. Seroconversion after vaccination was observed by day 7 post-vaccination in chronically infected herds, whereas in the case of silent infection, it started by day 14. These data indicated a pre-existing immunity in seronegative animals in chronically infected herds. Additionally, IFN-γ reactivity was detected in seronegative calves (>3 months) and heifers from chronically infected farms compared to a negative farm. An infection prior to 3 months of age resulted in cellular immunity in the absence of detectable antibodies. An infection around calving would explain this. The aforementioned circumscribed seronegative age groups are, therefore, explained by an infection early in life during active shedding at calving. Based on these results, an endemic cycle of coxiellosis is proposed: Susceptible young heifers get infected by persistently infected cows. Subsequently, shedding of *C. burnetii* at calving results in infection and then in cellular immunity in offspring. When these calves enter the cow herd two years later, a maximum of herd immunity is achieved, shedding ceases, and new susceptible animals are raised. In an acutely infected dairy farm, the PhI^+^/PhII^+^ serological pattern prevailed in second-lactation cows. In this study, stored sera collected since birth were analysed retrospectively. From the earliest seroconversion, the peak of seroconversion took about 33 months. These data suggested a slow spread of infection within herds. The classification of dairy cow herds is a promising basis for further analysis of the clinical impact of coxiellosis.

## 1. Introduction

The zoonotic pathogen *C. burnetii* is a small, Gram-negative obligate intracellular bacterium. Small ruminants (sheep, goats) are a major source of human Q fever [1]. In contrast, although dairy cow herds are frequently infected [2,3,4,5,6], at least in Europe, they are only sporadically associated with human Q fever [7]. Occupational obstetrics in cattle was determined as a major factor, at least for seroconversion of male veterinarians [8]. In contrast, in Australia, the majority of human cases were traced back to cattle [9].

*C. burnetii* is shed in a variety of excretions and secretions (urine, faeces, vaginal mucus, milk). Both persistent and intermittent shedding can be observed [10,11,12]. Because of its unique tropism for placental tissue, the highest concentrations of the pathogen are observed in the placenta and amniotic fluid [13,14]. Hence, infection spreads very efficiently during calving and even better when abortions occur directly in the herd. Long-term infectivity in the environment is preserved by spore-like (small cell variant) particles that may be easily spread by the wind [1,15]. In contrast, although *C. burnetii* was detected for prolonged time periods by PCR in environmental samples from a goat farm, viable *C. burnetii* were not detected in environmental samples 2, 3, or 4 months after the last *C. burnetii* positive parturition [16].

An analysis of the association of *C. burnetii* with reproductive disease in cattle concluded that data are still inconclusive due to the absence of a suitable case definition [17]. The presence of a complex of events (abortion, delivery of premature, stillborn, or weak calves) may indicate the involvement of *C. burnetii* [18]. An infection might result in placentitis [19], but *C. burnetii* shedding and mild lesions can also frequently be observed at normal calving [20]. The latter was explained by the fact that both the semi-allogenic foetus and *C. burnetii* are tolerated by the same immunological mechanism [21]. *C. burnetii* is frequently detected in milk. For individual cows, 10^1^–10^4^ bacteria/mL milk have been recorded [10,22], and animals with more than 10^3^ bacteria/mL milk were defined as high shedders [12]. In contrast, the identification of persistently infected animals is biased by a variable pattern of shedding (persistent, intermittent, and sporadic). Persistent and high-level shedding of *C. burnetii* is associated with a strong antibody response [11]. Therefore, an increased antibody reactivity is an additional valuable parameter for identifying persistently infected cows.

Serological diagnosis of Q fever in human medicine is based on the detection of antibodies against phase II (PhII), a protein antigen, and against phase I (PhI), primarily directed against full-length smooth lipopolysaccharide (LPS) [23,24,25]. PhII antibodies indicate a past or recent infection, whereas PhI antibodies, and especially increased PhI titres, are associated with chronic Q fever and a prolonged persistence of *C. burnetii* [24,26,27]. Regarding this different kinetics, it should be kept in mind that antibody responses against protein antigens (PhII) are generally T cell-dependent, whereas at least primary antibody responses to LPS are T cell-independent [28,29]. The clearance of intracellular bacteria requires a robust cellular immune response [23,30,31]. Consequently, the serological antibody pattern partially reflects the principal orientation of the immune response. In other words, an increased PhI antibody response, especially of the IgG1 subtype, suggests a failure of cellular immunity. Finally, it needs to be considered that PhI-LPS is one of two currently known virulence markers of *C. burnetii* [23,32]. Its binding to Toll-like receptor (TLR) 4 results in a reorganisation of filamentous actin in the cell wall and a reduction in the production of IFN-γ [33,34,35,36]. It has been hypothesised that PhI-LPS might be regarded as a TLR-4 antagonist [23,37,38].

Serological diagnosis in cattle still relies on complement fixation tests (CFT) or commercially available ELISAs coated with a mix of both PhI and PhII antigens originating from sheep, ticks, or cows [39]. However, earlier studies have demonstrated that the immune response to protein (i.e., PhII) and LPS (i.e., PhI) antigens is different in cattle after a natural infection versus vaccination: natural infection preferentially induces an IgG1-response both to proteins and LPS, whereas vaccination induces an early IgG2 response to the protein p27 of *C. burnetii*, as well as a late and weak IgG2 response to LPS [40,41,42].

One dairy herd was repeatedly analysed for PhI and PhII antibodies over time. At the herd level, the serological pattern PhI^−^/PhII^+^ in first-lactation cows was associated with the detection of *C. burnetii* in vaginal swabs collected at calving [3]. One year later, one-third of these cows remained in the same pattern (PhI^−^/PhII^+^), one-third switched to PhI^+^/PhII^+^, and the last third was characterised by a loss of antibodies. Five years later, the cows of this herd were repeatedly tested for antibodies and *C. burnetii* shedding, and it was demonstrated that a persistent infection was associated with elevated PhI titres [10]. Therefore, we hypothesised that endemic infections of dairy herds with *C. burnetii* follow a cycle.

The aim of this study was to provide data on the dynamics of the endemic cycle of coxiellosis in dairy farms. The following steps were taken to achieve these objectives. Firstly, using the measures of phase-specific antibodies and PCR detection of *C. burnetii* DNA in milk, four distinct herd profiles were recognised in a convenient sample of 49 Bavarian dairy farms. Secondly, by long-term analysis of one dairy herd over a period of four years, it was determined whether these profiles were cyclical. Thirdly, to clarify whether seronegative cows and heifers in infected herds are immune or susceptible, they were analysed for PhII-specific IFN-γ reactivity; it was also determined whether vaccination of seronegative cows resulted in an anamnestic antibody response on day 7 after vaccination. Fourthly, the time course of seroconversion in a group of second-parity cows was assessed retrospectively by analysis of stored sera that had been collected since birth. Finally, three exceptional cases with prevalent detection of *C. burnetii* in milk samples are presented.

## 2. Materials and Methods

### 2.1. Assessment of Antibody Profiles in 49 Bavarian Dairy Farms (Monitoring 2015)

In 2015, dairy producers were invited to assess the coxiellosis status of their herd. Farms with a history of vaccination against coxiellosis were excluded. Individual milk samples obtained from all lactating cows in the herd were tested for phase-specific antibodies, and if the farmer agreed, the samples were also tested for *C. burnetii* DNA by quantitative polymerase chain reaction (qPCR).

This approach resulted in milk from 2717 cows (771 primi- and 1946 multiparous cows) from 49 farms being analysed for phase-specific antibodies. The average number of lactating cows per farm was 55 (CI 95% 47–64, Min 23, Max 256). Of these, 23 herds (469 primi- and 1060 multiparous cows) were also tested by qPCR.

### 2.2. Long-Term Changes in the Antibody Profile in One Dairy Farm

In order to assess long-term changes in infection, annual testing of milk samples over a period of 4 years was performed in farm Kr (2014 n = 105, 2015 n = 110, 2016 n = 115 and 2017 n = 120). Farm Kr participated in the monitoring in 2015. This farm entered the study due to abortions of unknown origin in 2013/2014. Milk samples were tested by qPCR. The herd was not vaccinated against coxiellosis during the study.

### 2.3. The Immune Status of Seronegative Animals–Assessment of an Anamnestic Antibody Response in Seronegative Cows after Vaccination

Additional farms that were analysed after 2015 were included in this study to elucidate the dynamics of infection. Circumscribed seronegative age groups were frequently observed in infected dairy farms. In order to determine a pre-existing immunity in such animals, four dairy farms with varying seroprevalence according to an initial analysis were selected in 2022 (farm Br, G, Mr and Wn). Individual milk samples were collected the day prior to vaccination (Coxevac^®^, Ceva Sante Animal, Libourne, France) and 7 days later. Milk samples were analysed for phase-specific antibodies and by qPCR. Seroconversion in initially seronegative cows was determined on day 7 after vaccination. In farms Mr and Wn, additional samples were collected on days 14, 21 (i.e., at second vaccination), 28, and 56 after first vaccination.

### 2.4. The Immune Status of Seronegative Animals—IFN-γ Reactivity in Seronegative Heifers in Infected Farms

In the course of the control of vaccination in four farms in 2020 (farms Mz, Th, Wl and Z), the *C. burnetii*-specific interferon-γ (IFN-γ) response of seronegative heifers was determined. In farms Mz (82 cows), Th (132 cows), and Z (82 cows), infection was confirmed by individual milk testing (phase-specific antibodies and qPCR) of all lactating cows. In farm Wl (82 cows), serological screening of 30 individual milk samples revealed negative results for PhI and PhII antibodies; therefore, this farm was deemed to be non-infected at the time of testing. After the initial assessment of the herd status, cattle older than 3 months (Th, Z) and cattle older than 12 months (Mz and Wl) were vaccinated with Coxevac^®^. IFN-γ reactivity was determined after stimulation of whole blood with PhII antigen prior to first and after primary vaccination. The PhII-IFN-γ reactivity in seronegative animals was analysed prior to vaccination (Mz 10 cows, Th each 10 animals 3–6 and 7–12 months of age, Wl 14 heifers 12–25 months of age and 25 cows; and Z each 8 animals 3–6 and 12–15 months of age). Additionally, in farm Mz, 12 seropositive cows (PhI^+^/PhII^+^) with detection of *C. burnetii* at least once by qPCR were included as a control (Mz Ab^+^/qPCR^+^).

### 2.5. Time Course of Seroconversion in a Group of Cows in 2nd Lactation

Outbreaks of coxiellosis are frequently diagnosed, but information about the development of infection prior to such outbreaks is not available for routine submissions. Farm F is a federal research facility with about 110 dairy cows (Holstein Friesian, seasonal calving from September to January). *C. burnetii* was detected in January 2020 in one cow. The age group born in 2016 had already developed both PhI and PhII antibodies in April 2020. Stored sera (−80 °C) from this group were available at 1–4, 10–14, 18–22, 24–28, 35–39, and 42–46 months of age. These sera were retrospectively analysed for PhI and PhII antibodies in order to determine the time course of seroconversion.

### 2.6. Examples of Dairy Cow Herds with Prevalent Detection of C. burnetii in Milk Samples

In order to illustrate extreme cases, data from three farms (Ke 2022, Mj 2020, R 2021) were included (Mj 43 cows, mix of Holstein-Friesian/Bavarian Simmental, and Ke 101 cows, R 67 cows, both Bavarian Simmental). Individual milk samples were tested for phase-specific antibodies and by qPCR.

### 2.7. Vaccination

Vaccination against coxiellosis was generally proposed as an intervention measure, and animals were vaccinated with a commercial phase I vaccine (Coxevac^®^, Ceva Sante Animal, Libourne, France). The volume of 1 mL Coxevac^®^ contains ≥72 *C. burnetii* units (relative potency of phase I antigen measured by ELISA in comparison with a reference item) and is approximately equivalent to 100 µg of inactivated corpuscular phase I antigen of *C. burnetii*, according to the manufacturer. The vaccine was administered subcutaneously in a skinfold in front of the shoulder. Primary vaccination was performed according to the manufacturer’s instructions for cattle and consisted of two 4 mL doses three weeks apart.

### 2.8. Sample Collection

Trained technicians of the Bavarian Animal Health Services collected quarter milk samples aseptically (monitoring 2015, farm Kr (first sampling)). Milk was collected into sterile 9 mL vials containing 1 mL of 5% boric acid for preservation (Kabe Labortechnik GmbH, Nümbrecht, Germany). The samples were chilled immediately and shipped to the diagnostic department of the Bavarian Animal Health Service overnight.

Otherwise, farmers received 50 mL vials prepared with 0.05% sodium azide (sodium azide tablets 8 mg/tablet, Merck KgaA, Darmstadt, Germany) and were instructed about sample collection. Pooled milk from all quarters was collected into one vial after the cow had been prepared for milking.

The date of birth of each cow was provided by the farmer, and it was expressed as year and month of birth (YYMM).

Routine control of vaccination included blood sampling and/or milk sampling prior to and after vaccination. Li-Heparin blood samples (Primavette^®^ 9 mL, Kabe Labortechnik GmbH, Nümbrecht, Germany) and clotted blood samples (Monovette^®^ 9 mL Z, Sarstedt AG & Co. KG, Nümbrecht, Germany) were collected by jugular venepuncture. Blood samples were transferred directly to the laboratory at ambient temperature and were further processed in the IFN-γ restimulation assay the same day.

### 2.9. Tests

Phase-specific ELISA and IFN-γ restimulation assay (IFN-γ-RA) were performed as previously described [10,43].

Phase-specific ELISA: Skimmed milk was titrated 1/5, 1/50, 1/500, and 1/5000, and serum was titrated 1/100, 1/1000, 1/10,000, and 1/100,000 in sample diluent in PhI- and PhII-coated test plates. Bound antibodies were detected by a protein-G-peroxidase conjugate. A strong positive milk sample giving a maximum reaction served as positive control. The antibody titre was calculated as the reciprocal dilution at 20% optical density (OD) of the positive control. A titre of 5 (milk) and 100 (serum) scored negative. A PhI titre of ≥100 in milk was used as a screening for persistently infected cows. The serological phase pattern per cow was expressed as PhI^−^/PhII^−^, PhI^−^/PhII^+^, PhI^+^/PhII^−^, and PhI^+^/PhII^+^.

IFN-γ-RA: IFN-γ was assessed by stimulation of Li-heparinised blood in duplicate with PhII antigen, control antigen, PBS, and poke-weed mitogen (SC). Stimulation was performed in the presence of an IL-10-neutralising monoclonal antibody and an IgG1-isotype control. Here, only data with isotype control are presented. After 16 h of incubation at 37 °C, plasma was harvested after centrifugation and tested for IFN-γ (ELISA for bovine IFN-γ; Mabtech, Uppsala, Sweden). The concentration of IFN-γ (pg/mL) was calculated by a standard curve.

qPCR: For the detection and quantification of the multi-copy target (*C. burnetii* transposase gene, *IS1111*), a TaqMan^®^ real-time PCR (VetMAX™ C. burnetii Absolute Quant Kit, Thermo Fisher Scientific,/LSI, Lissieu, France) was used according to the manufacturer’s instructions. The DNA extraction was performed with a commercially available kit (IndiMag Pathogen Kit, Indical Bioscience GmbH, Leipzig, Germany) according to the manufacturer’s instructions.

### 2.10. Statistical Analysis

Phase-specific antibody profiles per herd were evaluated as follows: Year and month (YYMM) of birth were recorded for each animal. Cows within a given herd that were ≤12 months older than the youngest lactating cow were classified as primiparous/first-lactation cows. Animals were ordered by descending age; the youngest animals are shown in the figures in the Results section on the right side, covered by a grey bar. Profiles of PhI and PhII titres over age were analysed graphically per herd. PhI and PhII titres and the concentration of *C. burnetii* per mL milk are shown per animal, and additionally, to stress an increase in titres for distinct age groups, the moving average (n = 5 in herds with less than 100 cows and n = 10 in herds with more than 100 cows) of each titre is depicted as a fat line.

Statistical analysis of the data and preparation of profiles was performed with MedCalc^®^ version 19.1.2. (MedCalc Software, Broekestraat 52, B-9030 Mariakerke, Belgium). Generally, significance was assessed by a non-overlapping confidence interval of 95% (CI 95%). A comparison of IFN-γ reactivity was performed with ANOVA on log-transformed data. Pairwise comparisons of Ph patterns (Table 1 and Table 2) were performed by Fisher’s exact test with Bonferroni adjustment.

## 3. Results

### 3.1. Assessment of Antibody Profiles in 49 Bavarian Dairy Farms (Monitoring 2015)

The distribution of phase patterns in multi- and primiparous cows is summarised in Table 1. The proportions of PhI^−^/PhII^+^ and PhI^+^/PhII^+^ were similar; the PhI^+^/PhII^−^ pattern was rarely detected. Significantly more animals with elevated PhI titres (PhI ≥ 100) were observed in multiparous cows compared to primiparous cows.

In 23 dairy cow herds, the farmer agreed to allow detection of *C. burnetii* by qPCR. The overall rate of PCR-positive samples from multi- and primiparous cows was 4.2% (CI 95% 3.1–5.7%) and 1.9% (CI 95% 0.9–3.6%). The relationship between PCR-positive milk samples and the serological phase pattern is presented in Table 2. The detection of *C. burnetii* was significantly associated with PhI titre ≥ 100 both in multi- and primiparous cows. But, the detection of *C. burnetii* per phase pattern was not significantly different between multi- and primiparous cows.

**Figure 1:**
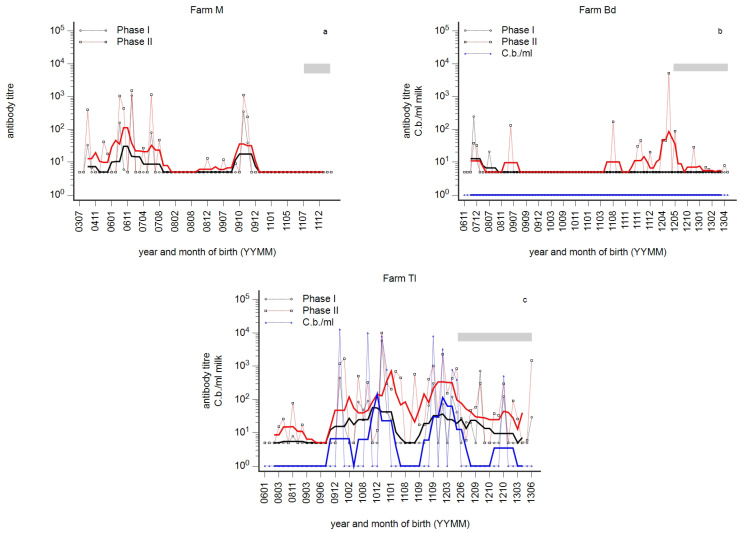
(**a**–**c**): Three prototypes of *C. burnetii* phase-specific antibody profiles. *C. burnetii* (blue), phase I (PhI, black), and phase II (PhII, red) antibodies were detected in milk samples from dairy cows. Three typical profiles by the year and month of birth (YYMM) are presented: silent (**a**), acute (**b**), and chronic state (**c**). The moving average is shown as a fat line, and n = 5 and n = 10 for herds with less than 100 cows and more than 100 cows, respectively. The grey bar indicates 1st lactating cows.

In order to assess age-related differences in antibody reactivity, the individual reactivity detected in milk was analysed by the age of cows. Three seronegative dairy farms were classified as likely to be free of infection. One of these herds was tested by qPCR; all milk samples tested negative. Otherwise, three distinct immune profiles in dairy cow herds were determined by the monitoring in 2015. The first type was characterised by the absence of antibodies in primiparous cows, while antibodies were detected in older cows (Figure 1a). This type was defined as the ‘silent state’ of infection at the herd level. The second type was characterised by PhII antibodies in primiparous cows, while PhI antibodies were not detected in this age group. The serological PhI^−^/PhII^+^ pattern in primiparous cows was defined as the ‘acute state’ infection at the herd level (Figure 1b). Remarkably, in many herds supposed to be acutely infected, antibodies were rarely detected in the next older age group to first-lactation cows, whereas antibodies were present again in older cows. This resulted in a typical wave-like pattern of antibody reactivity over age. Finally, the third type—the ‘chronic state’ of infection—was characterised by a PhI^+^/PhII^+^ pattern in primiparous cows (Figure 1c). In the silent, acute, and chronic states, antibodies were frequently detected in older animals.

Of all 49 tested herds, 3 herds were classified as seronegative (6.1%), 16 as in the silent (32.6%), 12 as in the acute (24.5%), and 18 as in the chronic (36.8%) state of herd infection. Aside from rarely observed seronegative herds, the silent, acute, and chronic states of herd-level infection were detected at an almost similar frequency.

### 3.2. Long-Term Changes in the Antibody Profile in One Dairy Farm

In order to assess whether these states occurred in a cyclic manner in a given herd over time, farm Kr was analysed annually from 2014 to 2017.

**Figure 2:**
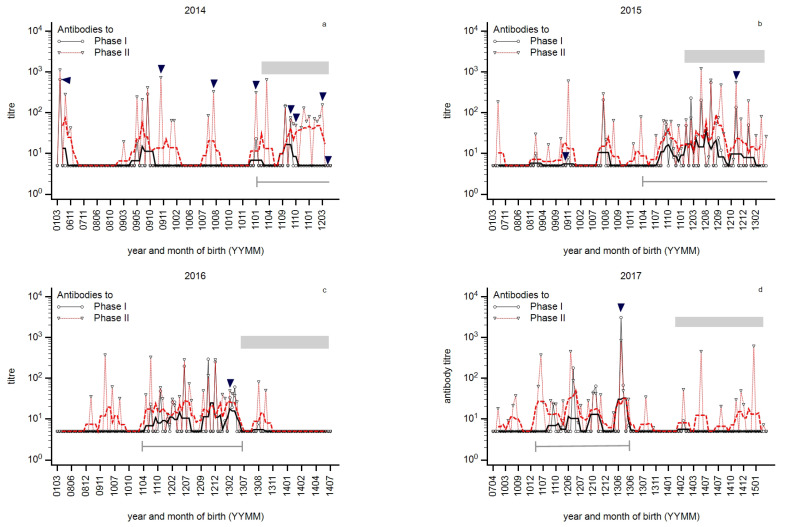
(**a**–**d**): Development of *C. burnetii* infection over a period of 4 years (herd Kr). PhI (black) and PhII (red) antibody profiles by the year and month of birth (YYMM) for 2014 (**a**), 2015 (**b**), 2016 (**c**), and 2017 (**d**). The moving average is shown as a fat line (n = 5). Animals with qPCR-positive milk samples are indicated by closed arrows. The horizontal bar indicates first-ion cows in 2014/2015; this bar moves due to aging of animals to the right. The grey bar indicates 1st-lactating cows in each year. (**a**) Upcoming PhI antibodies indicate the chronic state; however, it might be the late state of acute infection due to the difference between PhI and PhII titres. (**b**) Completely developed chronic state of infection. (**c**) Two cows in late first lactation with PhII titres might indicate the end or the start of a cycle. (**d**) The acute state of coxiellosis as indicated by PhII antibodies. A smooth transition from one state to the following state occurs.

The phase-specific profiles per year are presented in Figure 2a–d. The detection of PhII and PhI antibodies in 2014 indicated the chronic state of infection, although PhII antibodies were much more prevalent and detected at a higher level than PhI antibodies (Figure 2a). In 2015, both types of antibodies were prevalent in first-lactation cows, and similar titres of PhI and PhII antibodies were detected, i.e., the progressed chronic state of infection (Figure 2b). In 2016, the farm switched to a state close to the silent state as antibodies in first-lactation cows were merely detectable any longer. Only two cows at the end of first lactation tested positive for PhII antibodies (Figure 2c). Noteworthy, a group of older cows born in 2009/2010 showed a PhI^−^/PhII^+^ pattern. And finally, in 2017, the PhI^−^/PhII^+^ pattern was prevalent in first-lactation cows, indicating an acute infection (Figure 2d). A circumscribed group of young cows developed antibodies in 2014/2015 (indicated by a horizontal bar).

The antibody reactivity declined slightly over time. Notably, the next older age group to first-lactation cows in 2017 was characterised by the absence of detectable antibodies, although antibodies in first-lactation cows indicated an ongoing infection.

Cows in which *C. burnetii* had been detected in milk by qPCR are indicated by arrows. Data of selected cows are summarised in Table 3. No cow was repeatedly qPCR-positive; however, PhI titre ≥ 100 in two cows that had been tested only once suggested a persistent infection. Remarkably, the detection of *C. burnetii* was not always associated with seropositivity. In 2014, quarterly-based milk samples were tested by qPCR; not all quarters of the udder tested qPCR-positive.

### 3.3. The Immune Status of Seronegative Animals—Assessment of an Anamnestic Antibody Response in Seronegative Cows after Vaccination

**Figure 3:**
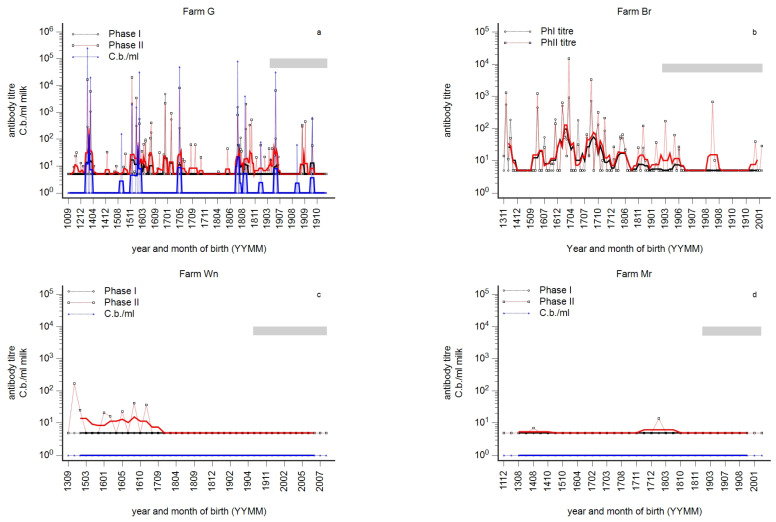
(**a**–**d**): Phase-specific antibody profiles of 4 dairy cow herds prior to vaccination against *C. burnetii*, in which the antibody response was assessed on day 7 after vaccination (Figure 4). Individual milk samples were tested for Phase I (black) and Phase II titres (red) and by qPCR (blue, *C. burnetii*/mL milk, except for farm Br). Individual results are presented by the year and month of birth (YYMM) of the cow. The moving average is shown as a fat line, and n = 5 and n = 10 for herds with less than 100 cows and more than 100 cows, respectively. The grey bar indicates first-lactation cows.

Well-circumscribed age groups of cows remained antibody-negative, although younger age groups seroconverted. In order to clarify whether these animals were immune or susceptible, we analysed the antibody response prior to first vaccination with Coxevac^®^ and 7 days later. Two infected (farms G and Br) and two dairy cow herds in the silent stage of infection (farms Mr and Wn) were included. The immune profiles of these herds at day 0 of vaccination are summarised in Figure 3a–d. A typical wave-like pattern was observed in farm G (Figure 3a). PhI^+^/PhII^+^ pattern in first-lactation cows indicated a chronic state of infection. Fourteen cows were qPCR-positive, and of these, nine cows showed a serological PhI^+^/PhII^+^ pattern with PhI titre ≥ 100. The geometric mean bacterial concentration was 14,678 C.b./mL (CI 95% 3169–67,975). One cow showed a PhI^−^/PhII^+^ pattern (63 C.b/mL), and four cows were seronegative (geometric mean 224 C.b./mL; CI 95% 10–5122).

An acute state and a typical wave-like pattern were assessed in farm Br. Similar PhI and PhII titres were observed in older cows. Antibodies were prevalent in cows born since the end of 2015 until mid-2019. PhII antibodies appeared in the youngest cows. The farmer refused the detection of *C. burnetii*. In farm Mr, only two cows showed a weak PhII titre, and in farm Wn, PhII but no PhI antibodies were detected only in older cows. Individual milk samples in both farms tested qPCR-negative. Both farms were classified to be in the silent state of infection.

**Figure 4:**
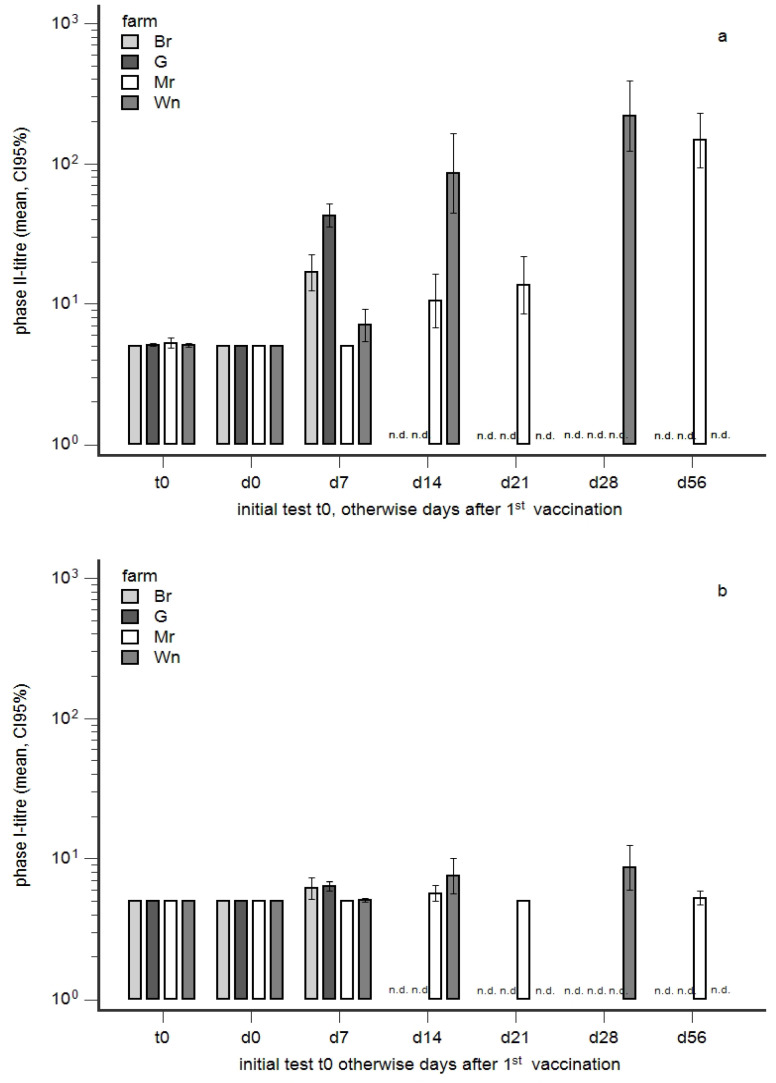
(**a**,**b**): Anamnestic antibody response in seronegative cows (milk) on day 7 after vaccination. Cows in farms G, Br, Mr, and Wn (Figure 3a–d) were vaccinated with Coxevac^®^. The development of phase II (**a**) and phase I (**b**) titres in seronegative cows on the day prior to 1st vaccination (d0) and days after 1st vaccination is presented. The initial testing of farms weeks prior to vaccination is indicated as t0. n.d. not done.

An anamnestic antibody response was detected in farms G and Br on day 7 after vaccination: PhII titres in seronegative cows significantly increased on day 7 post-vaccination as compared to day 0 (Figure 4a). In contrast, no significant increase in PhII titres was observed in seronegative cows in farms Mr and Wn on day 7, but PhII antibodies increased on day 14 and later. PhI antibody responses were not significantly different between herds and sampling d0/d7 (Figure 4b).

**Figure 5:**
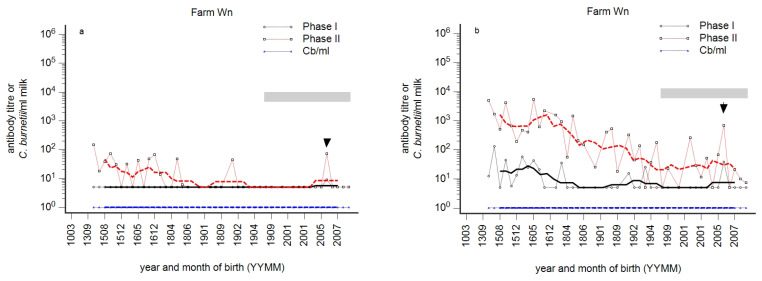
(**a**,**b**): Early development of phase-specific antibodies after vaccination. Cows in farm Wn (Figure 3c) were vaccinated with Coxevac^®^. Phase I (black) and Phase II (red) titres and detection of *C. burnetii* by qPCR (blue) at days 7 (**a**) and 14 (**b**) after vaccination are presented by the year and month of birth (YYMM) of the cow. The moving average (n = 5) is shown as a fat line. One cow (arrow) was bought from another farm. The grey bar indicates first-lactation cows.

In farm Wn, PhII antibodies were detected prior to vaccination in older cows, and susceptibility was expected in younger cows. Therefore, the development of antibodies over the age of animals on days 7 and 14 in farm Wn is depicted in Figure 5a,b, respectively. Seroconversion on day 7 was restricted to older cows, and in younger cows, PhII antibodies were detectable on day 14. Only one first-lactating cow seroconverted on day 7. This cow had been introduced from another farm.

### 3.4. The Immune Status of Seronegative Animals—IFN-γ Reactivity in Seronegative Heifers in Infected Farms

**Figure 6:**
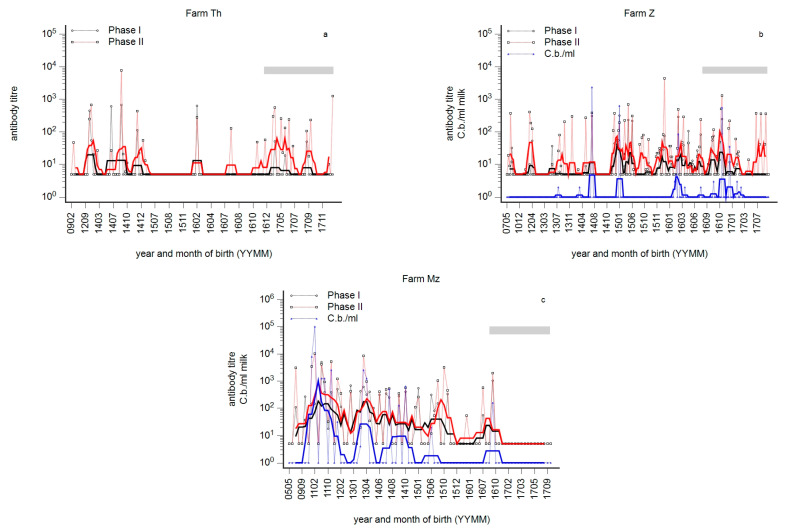
(**a**–**c**): Phase-specific profiles of 3 dairy cow herds in which interferon-γ reactivity was assessed in calves and heifers prior to vaccination against *C. burnetii* (Figure 7). Individual milk samples were tested for Phase I (black) and Phase II (red) titres and by qPCR (blue, *C. burnetii*/mL milk). Individual results are presented by year and month of birth (YYMM) of the cow. The moving average is shown as a fat line, and n = 5 and n = 10 for herds with less than 100 cows and more than 100 cows, respectively. The grey bar indicates first-lactation cows.

The phase-specific profiles of three herds prior to vaccination are summarised in Figure 6a–c. A fourth herd (Wl, not shown) was classified as seronegative prior to vaccination.

**Figure 7:**
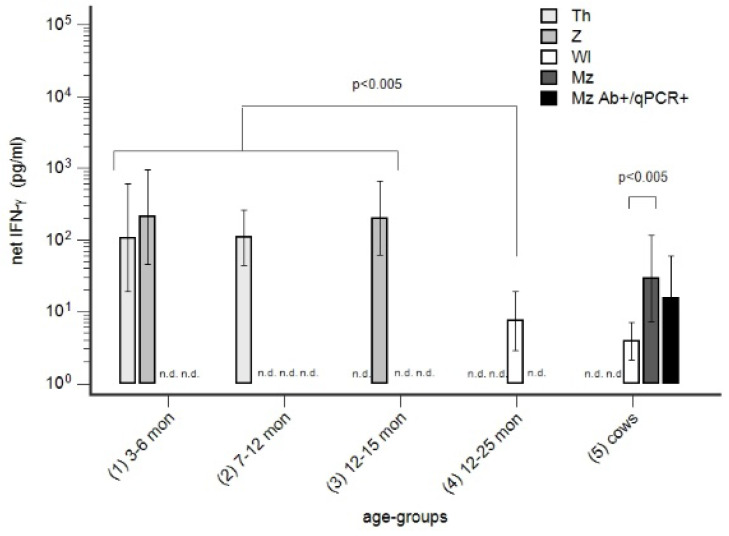
Interferon-γ (IFN-γ) reactivity in seronegative calves and heifers prior to vaccination against *C. burnetii* in infected farms. The vaccination against *C. burnetii* was controlled in infected farms Th, Z, and Mz (Figure 6a–c) and farm Wl, which was considered to be negative. The IFN-γ response prior to vaccination was assessed for seronegative animals of different ages. As a control in herd Mz, seropositive cows that were at least once qPCR-positive in milk were included (Mz Ab+/PCR+). n.d., not determined. n.d. non done.

The IFN-γ reactivity prior to vaccination was assessed in seronegative animals of different age groups (Figure 7). In infected herds (Th, Z), a significantly higher IFN-γ reactivity in animals 3–15 months of age was detected compared to animals 12–25 months of age in a dairy cow herd deemed to be negative (Wl). The IFN-γ reactivity of seronegative cows in the negative herd (Wl) was significantly lower compared to seronegative cows in an infected dairy farm (Mz). As a control, positive cows in farm Mz (Mz Ab^+^/qPCR^+^) were included. No significant difference in IFN-γ reactivity between seronegative and -positive cows in farm Mz was observed, but the IFN-γ reactivity in farm Wl was significantly lower.

### 3.5. Time Course of Seroconversion in a Group of Cows from Birth to Second Lactation

**Figure 8:**
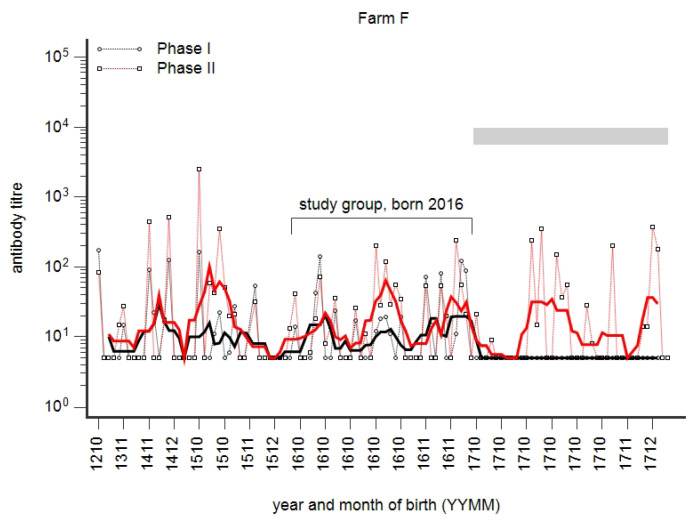
The phase-specific profile of a dairy farm (acute state of infection) prior to vaccination (April 2020) against *C. burnetii*. Individual milk samples were tested for Phase I (black) and Phase II (red) titres. Results are presented by the year and month of birth (YYMM). The moving average (n = 10) is shown as a fat line. The age group born in 2016 developed both PhI and PhII antibodies. The grey bar indicates first-lactation cows.

**Figure 9:**
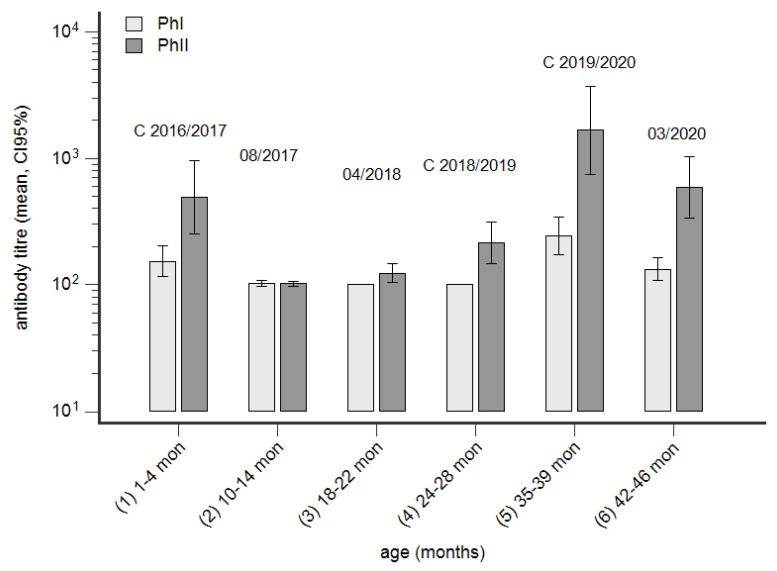
Development of seroconversion to *C. burnetii* from birth until third lactation. Phase I (PhI) and PhII antibodies had been detected in a group of cows born in 2016 (Figure 8). The time course of seroconversion since 2016 was retrospectively determined by stored serum samples. Calving season is indicated by “C”; otherwise, the month and year of sampling are presented.

The immune profile of cows in a research facility is shown in Figure 8. Based on the PhI^−^/PhII^+^ pattern in first-lactation cows, the herd was classified as acutely infected. The next older age group, born in 2016, was characterised by both PhI and PhII antibodies. The development of phase-specific antibodies in this age group from birth until 2020 is summarised in Figure 9. PhII reactivity was detected at the age of 1–4 months, and it decreased until 10–14 months of age. In April 2018, at the age of 18–22 months, a seroconversion was observed; it further increased at first calving in 2018/2019 and peaked at second calving in 2019/2020.

### 3.6. Examples of Dairy Cow Herds with Prevalent Detection of C. burnetii in Milk Samples

**Figure 10:**
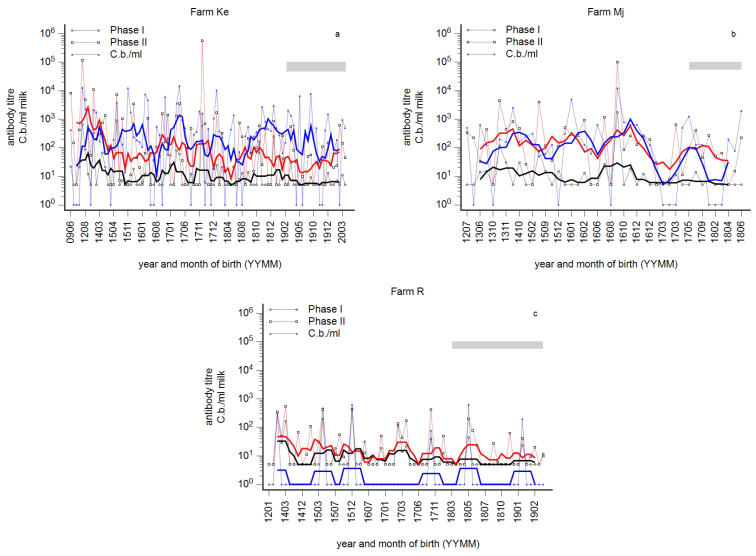
(**a**–**c**): Phase-specific profiles of three farms (Ke (**a**), Mj (**b**), R (**c**)) with a chronic state of infection and frequent detection of *C. burnetii* in milk samples. Individual milk samples were tested for Phase I (black) and Phase II (red) titres and by qPCR (blue, *C. burnetii*/mL milk). Individual results are presented by the year and month of birth (YYMM). The moving average is shown as a fat line, and n = 5 and n = 10 for herds with less than 100 cows and more than 100 cows, respectively. The grey bar indicates 1st-lactating cows.

Serological herd profiles are frequently characterised by a wave-like pattern, but in some cases, no wave-like pattern was observed. Examples are provided in Figure 10a–c. In two cases, numerous milk samples tested positive in qPCR. The detection of *C. burnetii* in milk samples was not associated with elevated PhI titres. In contrast, in the third case (Figure 10c), with no obvious wave-like pattern of the profile, only six cows were qPCR-positive. In three older cows, the detection of *C. burnetii* was associated with PhI titre ≥ 100, whereas in three young cows, the PhI titre remained below 100.

## 4. Discussion

Phase-specific serological tests and an IFN-γ-RA were established and applied in addition to pathogen detection by qPCR in routine diagnosis [3,10,43]. In order to get insights about the variability of antibody profiles, farmers were invited to submit milk samples from individual cows; these samples were tested for phase-specific antibodies and by qPCR, the latter only if the farmer agreed. In 2015, 49 farmers agreed to serological testing, but roughly 50% refused any qPCR testing because the detection of *C. burnetii* had to be reported to veterinary state authorities in Germany, and farmers were afraid of restrictions resulting from a positive test result.

Significantly more multiparous cows were positive for PhI antibodies compared to primiparous cows. Additionally, significantly more cows with elevated PhI titres ≥ 100, irrespective of parity, tested positive for *C. burnetii* by qPCR in milk. These data suggested that infection primarily affected primiparous cows. A higher rate of seroconversion in primiparous cows has been reported previously [44], and seroconversion in primiparous cows was further supported by the observation that heifers were frequently seronegative [45,46]. Thus, susceptibility in primiparous cows is an important prerequisite to maintaining infection at the herd level. Based on the simultaneous detection of *C. burnetii* and PhI ≥ 100 in milk, the prevalence of persistently infected cows was estimated to be about 2.3%. The true prevalence might be lower, as the farms were not randomly selected; farmers facing clinical problems showed a greater interest in participating.

Generally, *C. burnetii* was frequently detected in milk, e.g., 94, 84, and 56.6% of bulk-milk samples tested positive by qPCR in the USA, France, and The Netherlands, respectively [12,22,47]. The detection of *C. burnetii* by qPCR is not necessarily associated with infectivity, and infectivity of milk samples was demonstrated under laboratory conditions only. In one study of twenty-one raw milk samples, nine were qPCR-positive for *C. burnetii*. Subsequently, mice had been inoculated with six qPCR-positive highly concentrated fractions of these milk samples, and infectivity of *C. burnetii* was demonstrated after two passages in mice for two samples [4]. Other mice infection studies demonstrated that oral infection required high concentrations of *C. burnetii* (10^8^ bacteria) compared to aerosol infection [48]. Nevertheless, milk pasteurisation inactivated *C. burnetii* effectively [49], but the production and consumption of raw-milk products still bears a risk of infection. Therefore, the primary production of milk free of *C. burnetii* should be a major aim for the future.

### 4.1. The Different States of Herd-Level Infection

Phase-specific profiles by age of cows were analysed per herd, and an array of phase-specific profiles from routine diagnostic submissions was provided in this study. Three principal serological profiles were identified and provisionally designated as ‘acute’, ‘chronic’, or ‘silent’ states of infection (Figure 1). This provisional designation was based on two assumptions: The theory of phase-specific serology from human medicine was transferred to cattle, i.e., the PhI^−^/PhII^+^ pattern suggested an acute infection, and the additional appearance of PhI antibodies indicated a chronic course [27]. But, diagnosis of acute infection in humans also requires the specific detection of PhII-IgM [27]. This deficiency in experimental protocol was balanced by the detection of antibodies in first-lactation cows. The seroprevalence in nulliparous animals (>12 months) was very low or even undetectable; therefore, the detection of antibodies in first-lactation cows was interpreted as a recent seroconversion [3,46]. At the very least, this seroconversion probably takes place during the first two years of life, and the PhI^−^/PhII^+^ pattern in older cows does not necessarily indicate an acute herd infection (Figure 2c and Figure 6c). The additional detection of PhI antibodies was consequently characterised as the chronic state because PhI antibodies appeared later in the course of infection.

In addition to acutely and chronically infected herds, those with undetectable antibodies in primiparous cows were classified to be in the ‘silent’ state. The term ‘silent’ was used because older cows might have been seropositive or even qPCR-positive in milk due to persistence of *C. burnetii*. This profile expressed a decreased risk that *C. burnetii* was excreted at calving. Remarkably few dairy cow herds (n = 3) were considered free at the time of testing based on the inability to detect antibodies. In one of these herds, milk samples had been analysed by qPCR, and *C. burnetii* was not detected. As farms were not randomly selected, the rate of infection-free dairy cow herds might be underestimated. Therefore, in order to assess reliable rates per state of infection, a randomised study is required. However, the presented data are valuable in demonstrating the existence of different herd profiles.

### 4.2. Persistently Infected Cows

Persistently infected cows may provide a reservoir of *C. burnetii* in dairy cow herds. The detection of *C. burnetii* in individual milk samples was associated with PhI antibodies and even more pronounced with PhI titres ≥ 100 (Table 2). It had already been demonstrated that repeated detection of *C. burnetii* in cows was associated with elevated antibody reactivity and, more specifically, elevated PhI titres [10,11,50]. This situation was illustrated by profiles of farms G (Figure 3a) and Mz (Figure 6c), in which almost all qPCR-positive cows were classified as PhI titre ≥ 100. In contrast, in Figure 10a,b, two examples were presented in which *C. burnetii* was detected abundantly in milk samples and independently of the serological status. The lack of elevated PhI titres rather indicated an early state of infection in individual cows. Additionally, the absence of a wave-like pattern of the profile indicated that all cows might have been susceptible when *C. burnetii* was reintroduced to the herd.

Currently, the status of ‘persistently infected’ is difficult to assess, as repeated testing over years is required. Further, it has to be considered that *C. burnetii* might be detected in milk samples during acute infection; in some animals, it seems to persist for months after primary infection, and few cows even developed a prolonged persistence (years or even for life). This situation is further complicated by the possibility of intermittent shedding in persistently infected animals and by transient shedding in the course of re-infection. Consequently, the detection of *C. burnetii* alone is not sufficient for the decision to remove an infected cow from the herd. Therefore, an elevated PhI titre was an additional valuable criterion to identify persistently infected cows—e.g., a screening of milk samples for PhI titre ≥ 100 is suitable to narrow the number of milk samples that had to be retested by qPCR. Removing persistently infected cows from herds might be a promising control strategy. Notably, already in 1969, a German group achieved the eradication of infection in eight of nine herds by removing persistently infected cows from the herd [51]. In this study, milk samples had been inoculated in guinea pigs. However, the prevention of persistence might be far more effective.

The development of a persistent infection in individual cows is poorly understood. In humans, it was hypothesised that an increased number of apoptotic leucocytes due to endothelial lesions resulted in an M2 profile of macrophages/monocytes favouring the persistence of *C. burnetii* and the development of endocarditis [52,53]. Consequently, any physiological situation resulting in a dominance of the M2 profile of the innate immune system might favour the development of a persistent infection. Therefore, pregnancy and the periparturient period should be regarded as critical time periods in cattle [54,55]. Earlier, we could demonstrate the development of persistent infections in the course of the first lactation: *C. burnetii* was repeatedly detected in milk, PhI titres successively increased during first lactation, and three of four animals shed *C. burnetii* at the second calving; the fourth cow turned out to be non-pregnant [10]. Although *C. burnetii* was frequently detected in milk samples [56], the udder has to be considered as a route of primary infection. Schaal and Schaaf already postulated in 1969 that lactation might be a prerequisite for the development of persistent infection [51]. Bovine mammary gland epithelial cells had been infected in vitro with *C. burnetii* and were stimulated seven days later with *E. coli* LPS. Some strains of *C. burnetii* inhibited the pro-inflammatory response (IL-1ß, IL-6, TNFα) to *E. coli* LPS [57]. Based on this data, the question was raised if a primary infection through the teat canal, e.g., if a susceptible young cow followed a persistently infected cow during milking, initiated an unfavourable innate immune response that was subsequently enhanced or stabilised if *C. burnetii* spread to the pregnant uterus. Indeed, the infection is well tolerated during pregnancy [20,21]. Thus, clearance of infection might be primarily achieved in the time period between two pregnancies. But the non-pregnant time period per year in dairy cows is very short, and much shorter compared to small ruminants. This might increase the risk of dairy cows becoming persistently infected compared to small ruminants. Further, an aerogenic infection during pregnancy alone does not conclusively explain the development of a persistent infection in cows. Therefore, the route of primary infection (intranasal or intramammary) might be important. This view is further supported by studies performed on *Salmonella enterica* serovar Dublin: cows that were infected by the intramammary route and subsequently immunosuppressed developed a persistent infection of the udder [58].

In the case of *C. burneti*, susceptibility at the time of infection seemed to be a further prerequisite for the development of a persistent infection. Regarding the prevention of persistent infections, preliminary data indicated that a prophylactic vaccination prior to first breeding protected cows from becoming persistently infected (Böttcher, manuscript in preparation).

### 4.3. Susceptibility to Infection in Young Cows

Generally, undetectable antibodies are frequently mistaken for susceptibility to infection. Profiles like that illustrated in Figure 1b raised the question of why circumscribed age groups remained seronegative although younger cows obviously experienced an infection. Such a wave-like pattern of phase-specific profiles was repeatably seen (Figure 1a–c, Figure 2, Figure 3a–c and Figure 6a–c). Susceptibility of such seronegative animals in high-risk herds was excluded by the detection of αPhII-IFN-γ prior to vaccination (Figure 7 farm Mz) and by the assessment of an anamnestic immune response on day 7 after vaccination (Figure 4a). In contrast, seronegative animals in low-risk herds seroconverted on day 14 and later. These data suggested a higher likelihood of susceptibility, and—as illustrated in Figure 5a—especially in younger cows. An increased susceptibility in young cows explained a higher risk of seroconversion in primiparous cows [44]. The time course of seroconversion to phase-specific antigens was not yet determined in cattle; however, data in goats are available: a PhII and PhI seroconversion after infection of susceptible goats was detected after three and six weeks, respectively [59]. In summary, our data provided evidence that seronegative age groups in high-risk herds were likely to be immune. A similar situation was described for Q fever in humans: only 61 and 52% of skin test-positive people tested positive by immune fluorescent antibody test and complement fixation test, respectively [60,61]. Cellular immunity in the absence of humoral immunity was classically described for intracellular bacteria, of which mycobacterial infections like Johne’s disease or tuberculosis are prototypes [62,63]. Interestingly, even intradermal skin testing for tuberculosis resulted in anamnestic antibody response and increased the sensitivity of serological tests for *Mycobacterium bovis* [64]. Those seronegative age groups in our study did not even seroconvert despite an ongoing active infection in younger cows in the same herd (Figure 1b). This suggested that this type of immunity might be associated with a relatively high degree of protection. A pre-vaccination screening of calves and heifers revealed an αPhII-IFN-γ reactivity in seronegative calves and heifers at 3–12 months of age. Unfortunately, not exactly the same age groups of calves and heifers in affected and not-affected herds were tested, but nevertheless, these data suggested that infection might have occurred prior to the age of 3 months (Figure 7). Because *C. burnetii* was frequently detected at normal calving, we interpreted this as an infection around birth or even earlier during pregnancy [20]. Pre-colostral antibodies were not detected in calves, despite the detection of *C. burnetii* in vaginal swabs of the dams at calving [65,66]; therefore, IFN-γ testing of such calves is required to assess the proper immune status. Nevertheless, it is intriguing that an early acquired infection of calves with *C. burnetii* might have induced such a protective immune response. Therefore, further research on such animals is required to elucidate the parameters of protective immunity to *C. burnetii*. In contrast, an early infection of calves with *Mycobacterium avium* ssp. *paratuberculosis* established a persistent infection with an increased risk of clinical disease after a prolonged incubation period [62,63].

An improved efficacy of vaccination was described in seronegative cows and heifers in chronically infected farms after primary vaccination, i.e., two doses three weeks apart [67]. Our data suggested that this improved efficacy is rather explained by a pre-existing immunity in such seronegative cows. Consequently, concerns about the efficacy of vaccination in true susceptible animals were raised.

Finally, the question remains to be answered, how might true susceptibility be detected? Practically, an increasing number of seronegative young cows suggested an increased risk of susceptibility. This situation was illustrated by the post-vaccination immune response of seronegative cows in herd Wn (Figure 5). Thus, at the latest, prophylactic vaccination should be considered when a silent or a free state of infection is assessed.

If circumscribed seronegative age groups were explained by an immunity after early infection of calves, those antibody-positive age groups resulted from an infection in adulthood. Moreover, the width of such antibody waves in the profile mirrored the extent of susceptibility prior to infection of a dairy cow herd and thus mirrored the magnitude of a past outbreak. This view is supported by profiles in Figure 1c, Figure 6c and Figure 10a,b and the frequency of the detection of *C. burnetii* in milk. In other words, the prevalent detection of *C. burnetii* in milk samples in herds Ke and Mj (Figure 10a,b) was attributed to a high level of susceptibility prior to infection and assessment of the antibody response.

### 4.4. The Time Course of Infection

During the chronic state of infection, *C. burnetii* was detected abundantly in many types of samples [11,56]. As a snapshot of the situation in the herd and at first glance, this situation suggested high infectivity and a rapid spread of infection within herds, a situation generally termed as an ‘outbreak’. This view is further sustained by the frequently cited statement that even 1–10 bacteria might result in infection [68]. But so far, it has not been considered that the infection of the herd already might have started a long time previously. An example of how slowly infection progressed in a defined group of cattle was presented in Figure 9. The time from the first evidence of seroconversion until the peak of seroconversion was about 33 months; despite vaccination in April 2020, a few of these cows continued to shed *C. burnetii* at the third calving (Mertens-Scholz, personal communication). Consequently, the infection progressed for a long time unnoticed, probably at a low concentration, prior to its detection. Therefore, we suggested that the number of cows shedding *C. burnetii* at calving and the concentration of *C. burnetii* successively increased over time in the mode of a chain reaction until a critical threshold concentration of *C. burnetii* in the farm was reached. It seemed that this threshold value was not simply achieved by excretions and secretions like faeces, vaginal mucus, urine, and milk, and not at every parturition [20,69]. But high-level shedding at parturition, even of single cows, triggered the proposed chain reaction, finally leading to a major outbreak. Therefore, the length of pregnancy and seasonal calving primarily may have determined the prolonged time period of 33 months from the first seroconversion until the peak of the outbreak.

### 4.5. The Endemic Cycle

**Figure 11:**
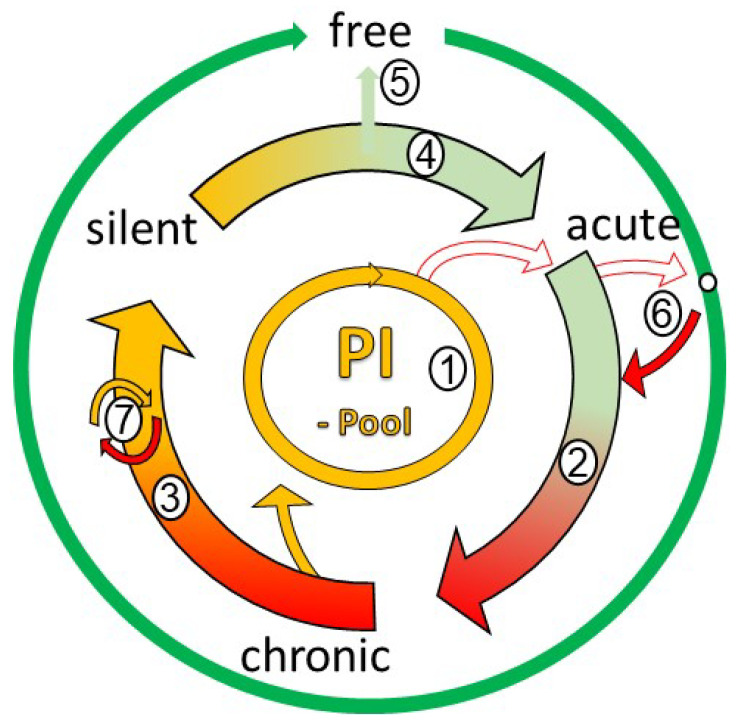
Endemic cycle of coxiellosis in dairy cow herds. Persistently infected cows (PIs) are central to the endemic cycle (1): They maintain long-term infection at the herd level and initiate new cycles of infection. In each cycle, the pool of PIs is replenished by a few cows that failed to control their infection in the course of the acute infection. The main states of herd-level infection (silent, acute, chronic) are shown as arrows (2–4). Herds might exit the cycle at the silent state to become free (5), e.g., if PIs had been removed, prophylactic vaccination was practised for years, and/or biosecurity measures were in place. The risk of major outbreaks increases with susceptibility (green). It is greatest for free herds (5) and increases at the end of the silent state. Infection of free herds is introduced from outside from acutely infected herds (6). Finally, some chronically infected herds might get arrested in the chronic state (7) if the dairy cow herd is constantly replenished with a sufficient number of susceptible primiparous cows, e.g., if only partial immunity is built up in calves due to good hygiene at calving and in the calf area.

The presented profiles were assessed in different dairy farms, and the question was raised as to whether these states were arranged into a cycle in a given herd. Indeed, we provided data that these states of infection successively occurred in one farm (Figure 2). Consequently, the endemic cycle of coxiellosis in dairy cow herds might be described as follows (Figure 11): Persistently infected cows shedding *C. burnetii* into milk or at calving are of central importance (Figure 11(1)) and are a common source of infection in many dairy farms [10,69]. They ensure the long-term persistence of *C. burnetii* in the herd, even during times of elevated herd immunity [10]. On the other side, a critical number of susceptible young cows appearing during the silent state of infection are required to initiate a new cycle (Figure 11(4)). In the case of primary acute infection (Figure 11(2)), bacteria are invading the pregnant uterus and *C. burnetii* is shed at the subsequent calving [21]. Shedding at calving and the concentration of *C. burnetii* at calving successively increase the environmental concentration of *C. burnetii*, finally resulting in an increased rate of infection and even re-infection of older cows [10]. Short/intermediate-term persistence of *C. burnetii* results in PhI antibodies, i.e., the chronic state of infection (Figure 11(3)). At this stage, the pool of PI animals is replenished in each cycle by acutely infected animals developing a persistent infection. An increased frequency and level of shedding of *C. burnetii* at calving induces a favourable cellular immunity without detectable antibodies in calves. When such immune calves enter the cow herd about two years later, herd immunity peaks, and the silent state of infection is achieved (Figure 11(4)). After the peak of immunity is reached, shedding at calving ceases. Calves do not get infected any longer. They are raised as susceptible animals, replenishing the pool of susceptible cows. Subsequently, the risk of a new cycle of infection increases with an increasing number of susceptible cows (Figure 11(4)). If no persistently infected animals are present anymore, dairy cow herds might switch to the free state (Figure 11(5)). In this case, *C. burnetii* has to be re-introduced from outside (Figure 11(6)). But generally, it has to be kept in mind that a re-infection by *C. burnetii* from outside might occur at any point in time, but persistently infected cows are a higher risk to initiate a new cycle. As the magnitude of an infection is determined by the number of susceptible cows, the risk of a major outbreak is greatest in free herds. As no wave-like pattern was present in herds Ke and Mj (Figure 10a,b), the frequent detection of *C. burnetii* in milk was explained by an infection of a free or almost-free dairy cow herd. No wave-like pattern was observed in farm R (Figure 10c), but the profile was nevertheless distinct from Ke and Mj: Firstly, in contrast to farms Ke and Mj, the moving average of PhI and PhII titres converged in farm R. Secondly, only a few cows tested positive by qPCR, of which the older cows presented as persistently infected (PCR^+^/PhI titre ≥ 100). Farm R was characterised by excellent hygiene and biosecurity. Very good calving hygiene (regular cleaning and disinfection of calving equipment and calving boxes, removal of the calf from the cow, separate housing of calves, etc.) is, therefore, suitable to prevent an efficient spread of infection of newborn calves. It prevents the development of early immunity in a proportion of calves; thus, a proportion of susceptible heifers is continuously reared and introduced to the cow herd, thereby maintaining active infection at the herd level. In other words, farms might get arrested in the chronic state (Figure 11(7)). This problem probably cannot be solved simply by increasing calving hygiene but may be ameliorated by the use of prophylactic vaccination of heifers against *C. burnetii* prior to their first breeding, which then decreases their susceptibility to coxiellosis.

### 4.6. The Endemic Cycle as a Basis for the Assessment of the Clinical Impact of Coxiellosis

Coxiellosis is generally associated with abortion, premature delivery, dead-borne calves and weak offspring [18]. Garcia-Ispierto and colleagues thoroughly reviewed the impact of coxiellosis on cattle health [17]: analysis of post-partum diseases like placenta retention, mastitis, metritis, and endometritis, the conception rate, and pregnancy loss were inconclusive. Neither the detection of *C. burnetii* nor the detection of antibodies were reliably associated with clinical disease. The latter was further biased by commercially available serological tests that do not differentiate the serological phase pattern because test plates are frequently coated with both antigenic phases [39]. Consequently, they concluded that studies on the clinical impact of coxiellosis were hampered by the absence of a proper case definition.

The detection of *C. burnetii* by qPCR might be regarded as a case definition. But, this classification is biased by the frequent detection of *C. burnetii* in apparently healthy cows [20]. The concentration of *C. burnetii* detected in a given animal might be considered a suitable classification variable, but the excreted concentration does not necessarily correlate to the concentration of the infecting dose. Moreover, it has to be kept in mind that the majority of infections in humans (e.g., 90% in the Dutch outbreak) were not associated with symptomatic disease [25,70,71]; a similar situation may be assumed for cattle.

An infection with *C. burnetii* frequently induces immunity; therefore, any case definition based on serological data is affected by immunity induced by a past infection [23]. Clinical disease might be expected in the case of recent seroconversion but not necessarily in seropositive immune animals. At least in guinea pigs and mice, the outcome of infection was dose-dependent; higher infecting doses are more likely to result in disease [72]. Additionally, the level of antibody titres also correlated with the infecting dose [72,73]. Based on these data, the antibody titre and phase pattern might be regarded as an appropriate classification. Under field conditions, it is impossible to determine the point-of-time of infection reliably. The PhI^−^/PhII^+^ pattern in older cows, as exemplified by the profiles in Figure 3c,d, was not necessarily attributable to an acute infection. But, our data indicated that an acute infection in first-lactation cows might be defined by phase-specific serology, and as infection primarily occurred during first lactation, the point-of-time of infection would be less variable. Consequently, any analysis of the impact of clinical disease should be focussed on first-lactation cows. Ideally, the antibody response has to be assessed after first and second calving in order to assess a seroconversion. This approach should be supplemented by the detection of *C. burnetii* at the first and second calving.

Currently, clinical disease is regarded as a direct effect of infection, but data on Q fever-fatigue syndrome (QFS) in humans, as observed in The Netherlands during the outbreak of 2007–2011, suggested that disease might present as a post-infection syndrome, too [74]. QFS is considered distinct from chronic Q fever because *C. burnetii* was not detected in affected people [75], whereas QFS has been attributed to an altered innate immune response after an infection with *C. burnetii* [75,76]. Notably, QFS was associated with an increased risk for upper respiratory disease [77], indicating that a past infection with *C. burnetii* might predispose to clinical disease associated with other infectious agents. These data raised the question as to whether similar long-term sequelae are of clinical importance in cattle, too. Therefore, any further analysis of the clinical impact of coxiellosis in dairy cow herds should also include such indirect effects of infection, too. Such sequelae are expected in circumscribed antibody-positive age groups, and the width of such antibody waves should be considered.

Farmers and veterinarians are extremely focussed on the clinical and economic impact of infectious diseases, and they are generally regarded as the basis for any justification of control programs. However, *C. burnetii* is a zoonotic pathogen classified as an agent of bioterrorism, and its presence in the milk of dairy cows is sufficient to argue for its control [23,78].

### 4.7. Practical Considerations

We analysed individual milk samples for phase-specific antibodies and for *C. burnetii* by qPCR. Milk samples have to be collected from all quarters of the udder because *C. burnetii* was not present in all quarters (Table 3). This had been reported earlier [51]. Therefore, composite milk samples per cow should be analysed.

Milk testing is influenced by colostrum quality of the sample, i.e., higher antibody titres were observed if colostrum was tested. This might result in false-positive results. Farmers should be advised to include only cows at least 3 days post-partum.

A further bias might result from newly introduced animals not raised in the farm. An example was provided in Figure 5: one young cow, which was bought from another farm, showed an antibody response on day 7 post-vaccination. Therefore, such cows should be indicated if profiles are analysed.

Additionally, in some farms in Bavaria (e.g., farm Mr, Figure 3d), cows are still kept in tie stalls and not in cubicle houses. It has to be kept in mind that infection dynamics are different in such holdings. The infection chain might be more easily interrupted at the early stage of infection, resulting in single seropositive animals. The risk of seropositivity increased with the size of the farm, suggesting a more efficient spread of infection in cubicle farms [3,79,80,81].

A slow development of infection over a prolonged time period was demonstrated in farm F (Figure 9). We explained this by a kind of chain reaction: successively more cows shed successively more *C. burnetii* at calving. This process might get interrupted at any time point, resulting in smaller antibody-positive age groups or even single or few seropositive animals. Practically, it should be considered that prophylactic vaccination is more effective at such early stages of infection in the presence of a low concentration of *C. burnetii*, as recently described for sheep [43,82].

Milk testing did not include dry cows. Persistently infected cows might be present in this group. But for the interpretation of the profile these cows are irrelevant.

Cows with increased PhI titres in first lactation are at risk of shedding *C. burnetii* at the next calving [10]. Biosecurity measures should be put in place to reduce any infection of humans.

IFN-γ testing was an interesting approach to elucidate the infection dynamics; however, it is not practical for routine diagnostics because blood samples had to be shipped within hours to the laboratory and had to be processed immediately after arrival at the laboratory. Additionally, it should be kept in mind that IFN-γ reactivity might decrease to an undetectable level after resolution of infection, which might result in false-negative results.

We analysed all cows for phase-specific antibodies. This kind of intensified testing is not affordable to farmers. Therefore, a simplified analysis should include cows in early and late first lactation and a bulk-milk sample. No detectable antibodies in these samples indicates a free herd. A positive bulk-milk sample and no detectable antibodies in first lactation define the silent state, PhII antibodies in first lactation indicate the acute state, and additionally detected PhI antibodies specify the chronic state of herd-level infection. As susceptibility has to be regarded as a major risk, dairy farmers with herds in the silent or free state of infection should be encouraged to implement a prophylactic vaccination program.

## 5. Conclusions

By analysing phase-specific antibody profiles of dairy cow herds, we identified an endemic cycle of coxiellosis and presented a panel of examples. Persistently infected cows shedding *C. burnetii* for a prolonged time or even for life maintained the infection at herd level, even in time periods of elevated herd immunity. The proportion of susceptible young cows in the herd determines the magnitude of subsequent infections. Shedding at calving ceased when the peak of herd-level immunity was achieved; it was a prerequisite for the production of a new generation of susceptible young animals. Removal of persistently infected cows and/or vaccination of pregnant heifers in order to reduce the susceptibility in young cows are therefore considered the most promising intervention measures. Completely susceptible herds are currently not desirable because of a high risk of re-infection.

## Figures and Tables

**Table 1:** ***Coxiella burnetii*-specific** antibody patterns in milk from dairy cows. Phase I and phase II antibody patterns (PhI/PhII), plus phase I antibody titres if ≥100, were determined in 2718 milk samples from 49 dairy cow herds in Bavaria, Germany. Serology patterns are compared in primiparous and multiparous cows.

	Multiparous Cows			Primiparous Cows		
Ph Pattern	n	%	CI 95%	n	%	CI 95%
PhI^−^/PhII^−^	1420	73.0	69.2–76.8	602	78.1	72.0–84.6
PhI^−^/PhII^+^	257	13.2	11.6–14.9	98	12.7	10.3–15.5
PhI^+^/PhII^−^	10	0.5	0.3–0.9	2	0.3	0.03–0.9
PhI^+^/PhII^+^	142	7.3	6.2–8.6	45	5.8	4.3–7.8
PhI ≥ 100	117	6.0	5.0–7.2	24	3.1	2.0–4.6

**Table 2:** Detection of DNA from *Coxiella burnetii* in milk (by qPCR) from cows and its relationship to the serological phase-pattern the milk. Milk samples (n = 1532) from 23 herds were examined.

	Multiparous Cows			Primiparous Cows		
Ph Pattern	n	% PCR^+^	CI 95%	n	% PCR^+^	CI 95%
PhI^−^/PhII^−^	709	0.14 ^a^*	0.0–1.8	336	0.00 ^α^*	0.0–1.1
PhI^−^/PhII^+^	162	0.60 ^a,b^	0.0–3.4	77	0.00 ^α,ß^	0.0–4.8
PhI^+^/PhII^−^	10	0.10 ^b,c,d^	0.25–55.7	1	0.00 ^ß,γ,δ^	0.0–100.0
PhI^+^/PhII^+^	103	11.7 ^c^	6.0–20.4	37	10.8 ^γ^	3.0–27.7
PhI ≥ 100	76	39.5 ^d^	26.6–56.5	18	27.8 ^δ^	9.0–64.8

* Values with different indices per column are significantly different (*p* < 0.05, Bonferroni-corrected).

**Table 3:** Changes over 4 years in the concentration in milk of DNA from *Coxiella burnetii* (by qPCR) on farm Kr. Comparison with serology (phase I and phase II) provided for 12 cows.

Animal	C.b./mL ^a^	Q n ^b^	PhI ^c^	PhII ^c^	C.b./mL ^a^	PhI ^c^	PhII ^c^	C.b./mL ^a^	PhI ^c^	PhII ^c^	C.b./mL ^a^	PhI ^c^	PhII ^c^
	2014				2015			2016			2017		
353	2.0	2	654	1099									
517	neg		5	5	2.4	5	5	neg	5	5			
525	1.7	2	5	719	neg	8	591	neg	5	371			
554	2.8	3	5	324	neg	5	5				neg	5	37
563	0.5	1	23	311									
585	1.5	2	65	74									
589	0.6	1	5	48	neg	8	13				neg	5	23
605	0.8	1	5	153	neg	231	72	neg	26	21			
606	0.5	1	5	5	neg	5	5	neg	5	5			
631					3.9	134	553	neg	253	278			
636					neg	5	27	2.8	33	48	neg	67	49
647								neg	19	42	2.2	3093	849

^a^ log_10_ *C. burnetii*/mL milk. ^b^ number of quarters/udder positive for *C. burnetii*. ^c^ Phase I and Phase II antibody titre.

## Data Availability

Data are unavailable due to privacy.
